# Abdominal obesity in type 1 diabetes associated with gender, cardiovascular risk factors and complications, and difficulties achieving treatment targets: a cross sectional study at a secondary care diabetes clinic

**DOI:** 10.1186/s40608-018-0193-5

**Published:** 2018-05-14

**Authors:** Eva O. Melin, Hans O. Thulesius, Magnus Hillman, Mona Landin-Olsson, Maria Thunander

**Affiliations:** 1 Department of Clinical Sciences, Section Endocrinology and Diabetes, Lund University, Lund, Sweden; 2 Department of Research and Development, Region Kronoberg, Box 1223, SE-35112 Växjö, Sweden; 3 Primary Care, Region Kronoberg, Växjö, Sweden; 4 Department of Clinical Sciences, Section of Family Medicine, Lund University, Malmö, Sweden; 5 Department of Clinical Sciences, Diabetes Research Laboratory, Faculty of Medicine, Lund University, Lund, Sweden; 6 Department of Endocrinology, Skane University Hospital, Lund, Sweden; 7 Department of Internal Medicine, Central Hospital, Växjö, Sweden

**Keywords:** Abdominal obesity, Cardiovascular complications, Diabetes mellitus type 1, Gender, Glycemic control, Hyperlipidemia, Hypertension, Inflammation, Treatment targets

## Abstract

**Background:**

Abdominal obesity is linked to cardiovascular diseases in type 1 diabetes (T1D). The primary aim was to explore associations between abdominal obesity and cardiovascular complications, metabolic and inflammatory factors. The secondary aim was to explore whether achieved recommended treatment targets differed between the obese and non-obese participants.

**Methods:**

Cross sectional study of 284 T1D patients (age 18–59 years, men 56%), consecutively recruited from one secondary care specialist diabetes clinic in Sweden. Anthropometrics, blood pressure, serum-lipids and high-sensitivity C-reactive protein (hs-CRP) were collected and supplemented with data from the patients’ medical records and from the Swedish National Diabetes Registry. Abdominal obesity was defined as waist circumference men/women (meters): ≥1.02/≥0.88. Hs-CRP was divided into low-, moderate-, and high-risk groups for future cardiovascular events (< 1, 1 to 3, and > 3 to ≤8.9 mg/l). Treatment targets were blood pressure ≤ 130/≤ 80, total cholesterol ≤4.5 mmol/l, LDL: ≤ 2.5 mmol/l, and HbA1c: ≤5 2 mmol/mol (≤ 6.9%). Different explanatory linear, logistic and ordinal regression models were elaborated for the associations, and calibrated and validated for goodness of fit with the data variables.

**Results:**

The prevalence of abdominal obesity was 49/284 (17%), men/women: 8%/29% (*P* < 0.001). Women (adjusted odds ratio (AOR) 6.5), cardiovascular complications (AOR 5.7), HbA1c > 70 mmol/mol (> 8.6%) (AOR 2.7), systolic blood pressure (per mm Hg) (AOR 1.05), and triglycerides (per mmol/l) (AOR 1.7), were associated with abdominal obesity. Sub analyses (*n* = 171), showed that abdominal obesity (AOR 5.3) and triglycerides (per mmol/l) (AOR 2.8) were associated with increasing risk levels of hs-CRP. Treatment targets were obtained for fewer patients with abdominal obesity for HbA1c (8% vs 21%, *P* = 0.044) and systolic blood pressure (51% vs 68%, *P* = 0.033). No patients with abdominal obesity reached all treatment targets compared to 8% in patients without abdominal obesity.

**Conclusions:**

Significant associations between abdominal obesity and gender, cardiovascular disease, and the cardiovascular risk factors low-grade inflammation, systolic blood pressure, high HbA1c, and triglycerides, were found in 284 T1D patients. Fewer patients with abdominal obesity reached the treatment targets for HbA1c and systolic blood pressure compared to the non-obese.

## Background

Both women and men with type 1 diabetes mellitus (T1D) have increased cardiovascular and all-cause mortality compared to persons without T1D, and the risk for premature death is increasing with increasing HbA1c levels [1]. Women with T1D are described to be at particular risk for both coronary artery calcification and for cardiovascular death across all age groups [1, 2]. The introduction of intensified insulin therapy for patients with type 1 diabetes mellitus (T1D) has led to decreased prevalence of diabetic retinopathy, nephropathy and neuropathy [3]. Intensive insulin therapy has however two major side effects, weight gain and increased frequency of severe episodes of hypoglycaemia [3]. Excess weight gain in T1D is associated with abdominal obesity, insulin resistance, dyslipidaemia, higher blood pressure, and atherosclerosis [4]. Particularly girls/women with T1D are at risk for developing overweight and obesity [5]. The prevalence of obesity is increasing globally [6]. When this study was conducted in 2009, the prevalence of general obesity (BMI ≥ 30 kg/m^2^) was 11% in men, and 10% in women in the general population in Sweden [7].

There is evidence that low-density lipoprotein (LDL) is both an indicator of future cardiovascular risk and a causal agent in the atherothrombotic process [8]. Raised triglycerides have been associated with low-grade inflammation, artery calcification, cardiovascular disease and all-cause mortality, and there is evidence that triglycerides are causal in the atherosclerosis process [9–12]. Common causes of raised triglycerides are obesity and high alcohol intake [9]. Impaired glycemic control has been linked to raised triglycerides in type 2 diabetes (T2D) [9]. Low levels of high-density lipoprotein (HDL) are strong predictors of atherosclerosis and cardiovascular disease [13]. However the causal relation between HDL and atherosclerosis is uncertain [13]. Lipid-lowering drugs are associated with a reduced risk of cardiovascular disease and death in T1D [14].

Chronic low-grade inflammation has been associated with obesity, insulin resistance, hypertension, hyperglycemia, acute hypoglycemia, dyslipidaemia, cardiovascular disease, and smoking [15–18]. One of the most frequently used markers of low-grade inflammation is high sensitivity C-reactive protein (hs-CRP), which is atherogenic, and a strong predictor of future cardiovascular events [15, 19]. Hs-CRP might be involved in mediating atherothrombotic disease through activation of complement pathways and immune cells [20].

In line with both international and Swedish national guidelines for diabetes, indications for lipid-lowering drugs at the clinic in 2009 were total cholesterol (TC) > 4.5 mmol/l or LDL > 2.5 mmol/l, in addition to dietary interventions and increased physical activity [21–23]. Indications for anti-hypertensive drugs were systolic blood pressure > 130 mmHg, or diastolic blood pressure > 80 mmHg [18, 21–24].

We have recently found that alexithymia, which is characterized by impaired capacity to identify and describe feelings, was associated with abdominal obesity [25]. We have also previously found that abdominal obesity, depression and smoking were independently associated with inadequate glycemic control [26].

The primary aim was to explore links between abdominal obesity, metabolic and inflammatory factors and cardiovascular complications in persons with T1D. The secondary goal was to explore whether obtained treatment targets for HbA1c, blood pressure, TC and LDL differed between the obese and non-obese participants.

## Methods

### Participants and procedures

**Fig. 1:**
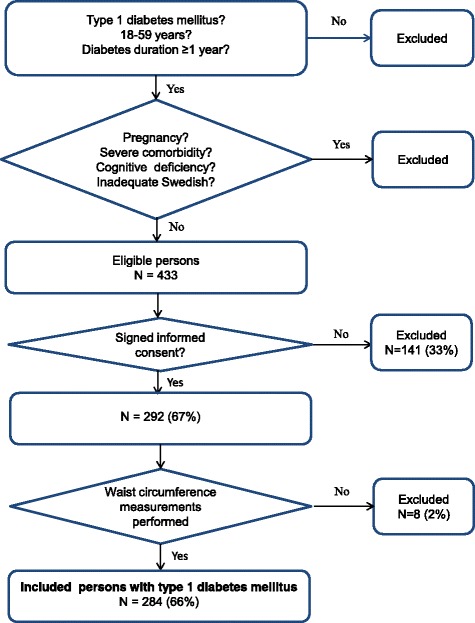
Description of criteria for inclusion in the study of obesity in persons with T1D

This study had a cross sectional design and was one of four baseline analyses [25–27] for a randomized controlled trial (ClinicalTrials.gov: NCT01714986) where “Affect School with Script Analysis” was tried against “Basic Body Awareness Therapy” for persons with diabetes, inadequate glycemic control and psychological symptoms [28, 29]. The participants were outpatients, consecutively recruited by specialist diabetes physicians or diabetes nurses, at regular follow up visits during the period 03/25/2009 to 12/28/2009. They were recruited from one secondary care specialist diabetes clinic, with a catchment population of 125,000 in southern Sweden. In this study 284 persons with T1D were included, 66% of the eligible patients (Fig. 1). Exclusion criteria were cancer, hepatic failure, end-stage renal disease, stroke with cognitive deficiency, psychotic disorder, bipolar disorder, severe personality disorder, severe substance abuse, or mental retardation. Anthropometrics, blood pressure and blood samples were collected. Data were collected from computerized medical records and the Swedish national diabetes register (S-NDR) [1, 23].

### Medication

Diabetes specific treatment was divided into three groups: multiple daily insulin injections (MDII), continuous subcutaneous insulin infusion (CSII), and MDII combined with oral antidiabetic agents (OAA) (ATC code A10BA02). The indications for OAA prescription in addition to insulin were obesity and insulin resistance.

Anti-hypertensive drugs were calcium antagonists (ATC codes C08CA01–02); angiotensin-converting enzyme (ACE) inhibitors (ATC codes C09AA-BA), angiotensin II antagonists (ATC codes C09CA-DA; diuretics (ATC code C03A); selective beta-adrenoreceptor antagonists (ATC code C07AB).

Lipid-lowering drugs were HMG CoA-reductase inhibitors (statins) (C10AA).

#### Anthropometrics and blood pressure

Waist circumference (WC), weight, length and blood pressure were measured according to standard procedures by a nurse. Abdominal obesity was defined as WC men/women (meters): ≥ 1.02/> 0.88 [30–32]. General obesity was defined as Body Mass Index (BMI): ≥ 30 kg/m^2^ for both genders [31].

#### HbA1c, serum-lipids and hs-CRP

HbA1c and serum lipids analyses were performed at the department of Clinical Chemistry, Växjö Central Hospital.

Venous HbA1c was analyzed with high pressure liquid chromatography, HPLC - variant II, Turbo analyzer (Bio – Rad®, Hercules, CA, USA). HbA1c > 70 mmol/mol (> 8.6%) corresponds to the 75th percentile in the whole population sample [26].

After an overnight fast, blood samples were collected and serum-lipids were were measured directly [8], using the enzymatic colour test (Olympus AU®, Tokyo, Japan). High TC was defined as > 4.5 mmol/l, high LDL as > 2.5 mmol/l, high triglycerides as ≥1.7 mmol/l; low HDL as < 1.04 mmol/l for men, and as < 1.29 mmol/l for women [33].

Samples for hs-CRP were collected, centrifuged, and stored at − 70 C Celsius until analyzed with spectrophotometry on a Roche Cobas C501 at the diabetes laboratory, Lund University Hospital, Lund. Hs-CRP was 0.54 ± 0.02 mg/l in healthy subjects according to previous research [16]. Hs-CRP < 1, 1 to 3, and > 3 to ≤10 mg/l correspond to low-, moderate- and high-risk groups for future cardiovascular events [19]. Samples with hs-CRP ≥10 mg/l were excluded as recommended in previous research [19]. Samples stored > 1 year were excluded. Hs-CRP was available for 171 (60%) participants.

### Treatment targets according to the Swedish National Guidelines for diabetes in 2009

The treatment targets recommend by the Swedish National Board of Health and Welfare were for T1D patients: 1) glycemic control: HbA1c ≤52 mmol/mol; 2) systolic/diastolic blood pressure: ≤130/≤80 mmHg; 3) serum-lipids: TC ≤4.5 and LDL ≤2.5 mmol/l [22].

### Hypoglycemia episodes

A severe hypoglycemia episode was defined as needing help from another person. Episodes during the last 6 months prior to recruitment were registered.

### Smoking and physical inactivity

Smokers were defined as having smoked any amount of tobacco during the last year.

Physical inactivity was defined as moderate activities, such as 30 min of walking, less than once a week.

### Cardiovascular complications

Cardiovascular complications were defined as ischemic heart disease or stroke/TIA.

#### Statistical analysis

Analysis of data distribution using histograms revealed that age, diabetes duration, hs-CRP, triglycerides, BMI and WC were not normally distributed. Data were presented as median values (quartile (q)_1_, q_3_; range), and analyses were performed with Mann-Whitney *U* test. Fisher’s exact test (two-tailed) and Linear-by-Linear Association (two-tailed) were used to analyze categorical data. Crude odds ratios (CORs) were calculated, variables with *P* ≤ 0.10, and age independent of *P*-value, were entered in multiple logistic regression analyses (Backward: Wald). The Hosmer and Lemeshow test for goodness-of-fit and Nagelkerke R^2^ were used to evaluate each multiple logistic regression analysis model. Ordinal regression analysis (stepwise forward) was performed with 3 risk levels of hs-CRP as dependent variables. Variables with *P*-values ≤0.10 in simple linear regression analyses were entered into multiple linear regression analyses (Backward). Confidence intervals (CIs) of 95% were used. *P* < 0.05 was considered statistically significant. SPSS® version 18 (IBM, Chicago, Illinois, USA) was used for statistical analyses.

## Results

In this population based cross sectional study of persons with T1D (*n* = 284, age 18–59 years, men 56%), persons with abdominal obesity (*n* = 49) were compared with non-obese persons (*n* = 235). Baseline data including comparisons between men and women are presented in Table 1. The women, compared with the men, had higher prevalence of both abdominal obesity (29% vs 8%, *P* < 0.001) and general obesity (18% vs 7%, *P* = 0.005). The men had higher systolic and diastolic blood pressure (both *P* < 0.001) and lower HDL (*P* = 0.002). The percentage that reached the recommended treatment targets were for HbA1c: 19%; TC: 48%; LDL: 36%; systolic blood pressure: 65%; diastolic blood pressure: 95%. Only 7% reached all treatment targets.

**Table 1:** Baseline characteristics and comparisons between men and women for 284 persons with T1D

	All patients	Men	Women	*P* ^a^
*N*	284	159 (56)	125 (44)	
Age (years)	42 (32, 51; 18–59)	43 (32, 52)	41 (30, 50)	0.12 ^b^
Diabetes duration (years)	20 (11, 30; 1–55)	21 (11–32)	19 (11, 29)	0.26 ^b^
WC (meters)	–	0.88 (0.82, 0.95; 0.65–1.33)	0.79 (0.75, 0.90; 0.63–1.25)	–
Abdominal obesity ^c^	49 (17)	13 (8)	36 (29)	< 0.001
General obesity ^d^	34 (12)	11 (7)	23 (18)	0.005
HbA1c > 52 mmol/mol (> 6.9%)	230 (81)	130 (82)	100 (80)	0.76
HbA1c > 70 mmol/mol (> 8.6%)	39 (24)	39 (24)	39 (31)	0.23
TC (mmol/l)	4.6 (4.1, 5.2; 2.1–10.9)	4.5 (4.0, 5.1)	4.7 (4.1, 5.4)	0.069 ^b^
High TC (> 4.5 mmol/l)	149 (52)	78 (49)	71 (57)	0.23
LDL (mmol/l)	2.8 (2.4, 3.3; 0.6–8.3)	2.8 (2.4, 3.3)	2.9 (2.4, 3.4)	0.51 ^b^
High LDL (> 2.5 mmol/l)	182 (64)	102 (64)	80 (64)	> 0.99
Triglycerides (mmol/l)	0.9 (0.7, 1.3; 0.6–5.9)	0.9 (0.7, 1.3)	0.8 (0.6, 1.3)	0.47 ^b^
High triglycerides (≥ 1.7 mmol/l)	34 (12)	16 (10)	18 (14)	0.28
HDL (mmol/l)	1.5 (1.3, 1.8; 0.8–2.7)	1.4 (1.2, 1.7)	1.6 (1.4, 1.8)	0.002 ^b^
Low HDL (M/W: < 1.04/< 1.29 mmol/l/)	32 (11)	17 (11)	15 (12)	0.85
Hs-CRP ^e^ (mg/l)	0.6 (0.3, 1.7; 0.03–8.9)	0.5 (0.2, 1.4)	0.9 (0.4, 2.5)	0.008 ^b^
SBP ^f^ (mm Hg)	120 (111, 130; 100–160)	125 (120, 130)	120 (110, 130)	< 0.001 ^b^
High SBP ^f^ (> 130 mmHg)	100 (35)	67 (42)	33 (26)	0.006
DBP ^h^ (mm Hg)	70 (70, 75; 55–100)	70 (70, 80)	70 (65, 75)	< 0.001 ^b^
High DBP ^g^ (> 80 mmHg)	13 (5)	10 (6)	3 (2)	0.16
Hypoglycemia (severe episodes)	13 (5%)	7 (4)	6 (5)	> 0.99
Smoking ^h^	28 (10)	18 (12)	10 (8)	0.42
Physical inactivity ^h^ (< 1/week)	31 (11)	18 (12)	13 (11)	0.85
CV ^i^ complications	10 (4)	6 (4)	4 (3)	> 0.99
LLD ^j^	133 (47)	77 (48)	56 (45)	0.55
AHD ^k^	95 (34)	61 (38)	34 (27)	0.057
MDII ^l^ and OAD ^m^	17 (6)	6 (4)	11 (9)	0.15
CSII ^n^	26 (9)	13 (8)	13 (10)	
MDII ^l^	241 (85)	140 (88)	101 (81)	
Reached all treatment targets ^o^	19 (7)	11 (7)	8 (6)	> 0.99

Data are n (%) or median (q_1_, q_3_; min-max)

^a^ Fisher’s exact test unless otherwise indicated. ^b^ Mann-Whitney *U* test. ^c^ WC: men/women ≥1.02/≥0.88 m. ^d^ BMI ≥30 kg/m^2^. ^e^ *N* = 171, missing values for men/women: *n* = 54/59. ^f^ Systolic blood pressure. ^g^ Diastolic blood pressure. ^h^ Missing values men/women: *n* = 6/6. ^i^ Cardiovascular. ^j^ Lipid-lowering drugs. ^k^ Anti-hypertensive drugs. ^l^ Multiple daily insulin injections. ^m^ Oral antidiabetic drugs. ^n^ Continuous subcutaneous insulin infusion. ^o^ Blood pressure ≤ 130/≤ 80, TC ≤ 4.5, LDL ≤ 2.5 and HbA1c ≤ 52

### Comparisons between patients with and without abdominal obesity

Results of comparisons between 49 persons with abdominal obesity and 235 persons without abdominal obesity are presented in Table 2. Persons with abdominal obesity had higher prevalence of HbA1c > 70 mmol/mol (> 8.6%) (*P* < 0.001), lipid-lowering drugs (*P* = 0.012) and cardiovascular complications (*P* = 0.016); and had higher median values of hs- CRP (*P* < 0.001), triglycerides (*P* < 0.001), systolic blood pressure (*P* = 0.004), LDL (*P =* 0.021) and TC (*P* = 0.047). Fewer patients with abdominal obesity compared to the non-obese reached the recommended treatment targets for HbA1c (8% vs 21%, *P* = 0.044) and systolic blood pressure (51% vs 68%, *P* = 0.033). No patients with abdominal obesity reached all risk factor treatment targets for blood pressure, TC, LDL and HbA1c compared to 8% in the non-obese.

**Table 2:** Comparisons between obese and non-obese, users and non-users of antihypertensive and lipid-lowering drugs

	Abdominal obesity			Anti-hypertensive drugs			Lipid-lowering drugs		
	Yes	No	*P* ^a^	Yes	No	*P* ^a^	Yes	No	*P* ^a^
*N*	49 (17)	235 (83)		95 (33)	189 (67)		133 (47)	151 (53)	
Age	45 (35, 53)	42 (31, 51)	0.11 ^b^	49 (42, 56)	39 (28, 56)	< 0.001 ^b^	49 (42, 54)	34 (27, 44)	< 0.001 ^b^
Diabetes duration	22 (14, 28)	20 (11, 31)	0.64^b^	29 (20, 35)	16 (9, 25)	< 0.001 ^b^	26 (14, 34)	17 (9, 24)	< 0.001 ^b^
Abdominal obesity	49 (100)	0		22 (23)	27 (14)	0.069	31 (23)	18 (12)	0.012
HbA1c > 52 mmol/mol (> 6.9%)	45 (92)	185 (79)	0.044	79 (83)	151 (80)	0.63	114 (86)	116 (77)	0.069
HbA1c > 70 mmol/mol (> 8.6%)	24 (49)	54 (23)	< 0.001	29 (30)	49 (26)	0.48	40 (30)	38 (25)	0.42
TC (mmol/l)	4.7 (4.2, 5.8)	4.6 (4.1, 5.1)	0.047 ^b^	–	–	–	4.5 (4.0, 5.2)	4.6 (4.1, 5.2)	0.32 ^b^
High TC (> 4.5 mmol/l)	29 (59)	120 (51)	0.35	–	–	–	63 (47)	86 (57)	0.12
Triglycerides (mmol/l)	1.2 (0.8, 1.9)	0.9 (0.7, 1.1)	< 0.001 ^b^	–	–	–	1.0 (0.7, 1.3)	0.8 (0.7, 1.1)	0.014 ^b^
High triglycerides (≥ 1.7 mmol/l)	14 (29)	20 (8)	< 0.001	–	–	–	21 (16)	13 (9)	0.070
HDL (mmol/l)	1.5 (1.3, 1.7)	1.5 (1.3, 1.8)	0.47 ^b^	–	–	–	1.5 (1.3, 1.8)	1.5 (1.3, 1.8)	0.48 ^b^
Low HDL (m/w:< 1.04/1.29 mmol/l)	5 (10)	27 (11)	> 0.99	–	–	–	12 (9)	20 (13)	0.35
LDL (mmol/l)	3.2 (2.5, 3.8)	2.8 (2.4, 3.3)	0.021 ^b^	–	–	–	2.7 (2.3, 3.3)	3.0 (2.5, 3.4)	0.058 ^b^
High LDL (> 2.5 mmol/l)	36 (74)	146 (62)	0.14	–	–	–	74 (56)	108 (72)	0.006
Hs-CRP ^c^ (mg/l)	2.5 (0.6, 4.6)	0.6 (0.2, 1.4)	< 0.001 ^b^	0.6 (0.3, 1.7)	0.7 (0.3, 1.9)	0.87	0.6 (0.3, 1.8)	0.8 (0.3, 1.7)	0.97
SBP (mm Hg)	130 (120, 132)	120 (110, 130)	0.004 ^b^	130 (125, 135)	120 (110, 125)	< 0.001 ^b^	–	–	–
High SBP (> 130 mmHg)	24 (49)	76 (32)	0.033	59 (62)	41 (22)	< 0.001	–	–	–
DBP (mm Hg)	70 (70, 78)	70 (65, 75)	0.051 ^b^	70 (70, 78)	70 (65, 75)	0.011 ^b^	–	–	–
High DBP (> 80 mmHg)	5 (10)	8 (3)	0.054	7 (7)	6 (3)	0.14	–	–	–
Hypoglycemia (severe episodes)	3 (6)	10 (4)	0.48	–	–	–	–	–	–
Smoking	4 (9)	24 (11)	0.80	6 (6)	22 (12)	0.20	13 (10)	15 (10)	> 0.99
Physical inactivity (< 1/week)	9 (19)	22 (10)	0.084	10 (11)	21 (12)	> 0.99	11 (8)	20 (14)	0.18
CV complications	5 (10)	5 (2)	0.016	7 (7)	3 (2)	0.018	9 (7)	1 (1)	0.007
LLD	31 (63)	102 (43)	0.012	62 (65)	71 (38)	< 0.001	–	–	–
AHD	22 (45)	73 (31)	0.069	–	–	–	62 (47)	33 (22)	< 0.001
MDII and OAD	13 (27)	4 (2)	< 0.001	–	–	–	–	–	–
CSII	1 (2)	25 (10)		–	–	–	–	–	–
MDII	35 (71)	206 (88)		–	–	–	–	–	–
Reached all treatment targets	0	19 (8)	0.052	–	–	–	–	–	–

Data are n (%) or median (q_1_, q_3_)

^a^ Fisher’s exact test unless otherwise indicated. ^b^ Mann-Whitney *U* test. ^c^ Missing values for abdominal obesity/no abdominal obesity: *N* = 27 (55%)/86 (37%)

### Comparisons between users and non-users of anti-hypertensive and lipid-lowering drugs

Persons treated with anti-hypertensive drugs had higher prevalence of high systolic blood pressure (62% vs 22%, *P* < 0.001), and cardiovascular complications (*P* = 0.018) (Table 2). Patients treated with lipid-lowering drugs had significantly higher median triglycerides (*P* = 0.014), higher prevalence of cardiovascular complications (*P* = 0.007), and lower prevalence of high LDL (*P* = 0.006) than non-users of lipid-lowering drugs (Table 2).

### Factors associated with abdominal obesity

Women (adjusted odds ratio (AOR) 6.5), systolic blood pressure (per mm Hg) (AOR 1.05), HbA1c > 70 mmol/mol (> 8.6%) (AOR 2.7), triglycerides (per mmol/l) (AOR 1.7), and cardiovascular complications (AOR 5.7) were associated with abdominal obesity (Table 3). Gender analyses showed that diastolic blood pressure (per mm Hg) (AOR 1.13) and anti-hypertensive drugs (AOR 5.3) were associated with abdominal obesity in men. Triglycerides (per mmol/l) (AOR 2.1), lipid-lowering drugs (AOR 3.1), and HbA1c > 70 mmol/mol (> 8.6%) (AOR 2.9), were associated with abdominal obesity in women.

**Table 3:** Associations with abdominal obesity in patients with T1DM, presented for all and gender specified

	Abdominal obesity							
	Both genders				Men		Women	
	*N* = 284 ^a^		*N* = 272		*N* = 158		*N* = 119	
	COR	*P*	AOR	*P* ^b^	AOR	*P* ^b^	AOR	*P* ^b^
Gender (women)	4.5 (2.3–9.0)	< 0.001	6.5 (2.9–14.5)	< 0.001	–	–	–	–
Age (per year)	1.02 (1.00–1.05)	0.11	1.01 (0.97–1.06)	0.54	1.03 (0.97–1.10)	0.31	1.01 (0.96–1.06)	0.78
Diabetes duration (per year)	1.00 (0.98–1.03)	0.77	–	–	–	–	–	–
HbA1c > 70 mmol/mol (> 8.6%)	3.2 (1.7–6.1)	< 0.001	2.7 (1.3–5.7)	0.009	1.9 (0.5–7.1)	0.36	2.9 (1.2–7.2)	0.022
TC	1.2 (0.9–1.6)	0.15	–	–	–	–	–	–
Triglycerides (per mmol/l)	1.9 (1.3–2.8)	< 0.001	1.7 (1.1–2.6)	0.010	1.0 (0.5–2.0)	> 0.99	2.1 (1.2–3.7)	0.011
HDL (per mmol/l)	0.7 (0.3–1.6)	0.34	–	–	–	–	0.4 (0.1–1.7)	0.24
LDL (per mmol/l)	1.4 (1.0–1.9)	0.072	1.0 (0.7–1.70)	0.86	–	–	1.0 (0.56–1.98)	0.88
SBP (per mm Hg)	1.04 (1.01–1.07)	0.004	1.05 (1.01–1.08)	0.005	1.01 (0.94–1.10)	0.72	1.03 (0.99–1 .07)	0.20
DBP (per mm Hg)	1.04 (1.00–1.09)	0.045	1.02 (0.96–1.09)	0.47	1.13 (1.03–1.24)	0.007	–	–
Hypoglycemia	1.5 (0.4–5.5)	0.57	–	–	–	–	–	–
Smoking ^c^	0.8 (0.3–2.4)	0.66	–	–	–	–	–	–
Physical inactivity	2.1 (0,9–4.8)	0.083	1.7 (0.5–5.2)	0.36	–	–	3.4 (0.8–14.4)	0.10
CV complications	5.2 (1.5–18.9)	0.011	5.7 (1.1–28.9	0.035	–	–	–	–
LLD	2.2 (1.2–4.2)	0.013	1.9 (0.9–4.1)	0.096	–	–	3.1 (1.3–7.6)	0.014
AHD	1.8 (1.0–3.4)	0.064	1.1 (0.5–2.6)	0.79	5.3 (1.3–20.7)	0.018	–	–

^a^ Unless indicated. ^b^ Multiple logistic regression analysis (Backward: Wald). ^c^ Missing values: *n* = 12. All/men/women: Hosmer and Lemeshow: Test 0.039/0.799/0.471; Nagelkerke R Square 0.335/0.234/0.250

### Factors associated with high-, moderate- and low-risk hs-CRP levels in 171 persons

Abdominal obesity (AOR (CI) 5.3 (2.1–13.6)) and triglycerides (per mmol/l) (AOR (CI) 2.82 (1.68–4.93)) were associated with increasing risk levels of hs-CRP (Table 4).

**Table 4:** Associations with low-, moderate- and high-risk hs-CRP levels

			Hs-CRP risk levels							
		All (With CRP)	Low (< 1 mg/l)	Moderate (1 to ≤3 mg/l)	High (> 3.0 to ≤ 8.9 mg/l)		Increasing hs-CRP risk levels			
		*N* = 171	*N* = 107	*N* = 44	*N* = 20		*N* = 171			
		N (%) or median (q_1_, q_3_)				*P* ^*a*^	COR (CI)	*P* ^*b*^	AOR (CI)	*P* ^*b*^
Gender	Women	66 (39)	35 (33)	19 (43)	12 (60)	0.017	2.0 (1.1–3.8)	0.023	–	NS
	Men	105 (61)	72 (67)	25 (57)	8 (40)					
Age		42 (30, 50)	42 (31, 50)	40 (27, 51)	42 (29, 53)	0.73 ^c^	1.00 (0.97–1.02)	0.72	–	NS
Abdominal obesity		22 (13)	7 (6)	5 (11)	10 (50)	< 0.001	7.0 (2.8–17.9)	< 0.001	5.3 (2.1–13.6)	< 0.001
HbA1c > 70 mmol/mol		51 (30)	27 (25)	11 (25)	13 (65)	0.004	2.2 (1.2–4.3)	0.016	–	NS
TC (mmol/l)		4.6 (4.1, 5.2)	4.5 (4.0, 5.1)	4.8 (4.1, 5.4)	4.8 (4.4, 6.0)	0.035 ^c^	1.5 (1.2–2.0)	0.002	–	NS
Triglycerides (mmol/l)		0.9 (0.7, 5.2)	0.8 (0.6, 1.1)	1.0 (0.7, 1.4)	1.3 (0.9, 2.4)	< 0.001 ^c^	3.2 (1.9–5.7)	< 0.001	2.82 (1.68–4.93)	< 0.001
HDL (mmol/l)		1.5 (1.3, 1.8)	1.6 (1.3, 1.8)	1.6 (1.3, 1.7)	1.4 (1.1, 1.7)	0.32 ^c^	0.6 (0.2–1.4)	0.21	–	NS
LDL (mmol/l)		2.9 (2.3, 3.3)	2.7 (2.2, 3.2)	2.9 (2.4, 3.6)	3.2 (2.8, 3.6)	0.010 ^c^	1.7 (1.2–2.4)	0.002	–	NS
SBP (mm Hg)		120 (115, 130)	120 (115, 130)	120 (110, 130)	128 (120, 134)	0.16 ^c^	1.02 (0.99–1.05)	0.15	–	NS
DBP (mm Hg)		70 (70, 75)	70 (65, 75)	70 (66, 80)	75 (70, 78)	0.036 ^c^	1.07 (1.02–1.12)	0.003	–	NS
LLD		82 (48)	50 (47)	21 (48)	11 (55)	0.55	1.2 (0.6–2.2)	0,60	–	NS
AHD		51 (30)	31 (29)	14 (32)	6 (30)	0.82	1.1 (0.6–2.1)	0.78	–	NS
Smoking ^d^		18 (10)	11 (10)	5 (12)	2 (10)	0.96	–	–	–	–
Physical inactivity ^e^		22 (13)	10 (10)	10 (23)	2 (10)	0.28	–	–	–	–
CV complications		7 (4)	4 (4)	0	3 (15)	0.16	2.3 (0.4–12.1)	0.30	–	NS

*NS* Non-significant

^a^ Linear-by-linear Association (Exact 2-sided) unless indicated. ^b^ Ordinal regression analyses. ^c^ Kruskal-Wallis test. Missing values: ^d^ *n* = 2; ^e^ *n* = 3. ^d, e^ Not included in the ordinal regression analyses

### Factors associated with gender, high HbA1c, systolic blood pressure and cardiovascular complications

Positive associations with women were found for abdominal obesity AOR 8.6 (3.9–19.0), *P* < 0.001; and HDL (per mmol/l) AOR 6.1 (2.7–13.6), *P* < 0.001. Negative associations with women were found for diastolic blood pressure (per mm Hg) AOR 0.91 (0.87–0.95), *P* < 0.001; and age (per year) AOR 0.97 (0.94–0.99), *P* = 0.005. Systolic blood pressure, TC and anti-hypertensive drugs were not associated with women (all *P* >  0.21). Nagelkerke R Square: 0.277. Hosmer and Lemeshow Test: 0.034.

The associations with HbA1c > 70 mmol/mol (> 8.6%) were for abdominal obesity AOR 2.7 (1.4–5.4), *P* = 0.004; triglycerides (per mmol/l) AOR 1.7 (1.1–2.5), *P* = 0.010; and for diastolic blood pressure (per mm Hg) AOR 1.04 (1.00–1.08), *P* = 0.090. HDL, TC, age, physical inactivity, and LDL were not associated with HbA1c > 70 mmol/mol (all *P* >  0.16). Nagelkerke R Square: 0.137. Hosmer and Lemeshow Test: 0.782.

The B-coefficients for the associations with systolic blood pressure were for age 0.24 (0.13–0.34), *P* < 0.001; anti-hypertensive drugs 6.5 (3.9–9.2), *P* < 0.001; triglycerides (per mmol/l) 2.0 (0.4–3.6), *P* = 0.014; men 4.0 (1.6–6.4), *P* = 0.001; and for abdominal obesity 4.0 (0.8–7.3), *P* = 0.014*.* Lipid-lowering drugs (*P* = 0.73) and diabetes duration (*P* = 0.99) were not associated with systolic blood pressure. Adjusted R Square 0.276, *P* < 0.001.

Associations with cardiovascular complications were for age (per year) AOR 1.18 (1.05–1.32), *P* = 0.006; abdominal obesity AOR 5.5 (1.4–22.0), *P* = 0.017; and for LDL (per mmol/l) AOR 0.3 (0.1–1.1), *P* = 0.071. Lipid-lowering drugs, anti-hypertensive drugs and diabetes duration were not associated with cardiovascular complications (all *P* > 0.34). Nagelkerke R Square: 0.309. Hosmer and Lemeshow Test: 0.978.

### Comparisons of patients with and without CRP measurements – A response analysis

The prevalence of abdominal obesity was lower in the 171 patients with hs-CRP measurements than in the patients without hs-CRP measurements (13% vs 24%, *P* = 0.024). Otherwise, they did not differ by medians for age (*P* = 0.10), diabetes duration (*P* = 0.52), diastolic blood pressure (*P* = 0.52), systolic blood pressure (*P* = 0.66), HDL (*P* = 0.49), LDL (*P* = 0.50), triglycerides (*P* = 0.70), TC (*P* = 0.79); or by prevalence of anti-hypertensive drugs (*P* = 0.124), physical inactivity (*P* = 0.33), severe hypoglycemia episodes (*P* = 0.38), HbA1c > 70 mmol/mol (*P* = 0.48), lipid-lowering drugs (*P* = 0.72), cardiovascular complications (*P* = 0.74), or smoking (*P* = 0.84).

## Discussion

In this cross-sectional study of abdominal obesity in 284 persons with T1D, age 18–59 years, consecutively recruited from one secondary care specialist diabetes clinic, we found that cardiovascular complications, women, increasing risk levels of hs-CRP, systolic blood pressure, marked inadequate glycemic control (HbA1c > 70 mmol/mol), and triglycerides were independently associated with abdominal obesity. Inadequate glycemic control, systolic blood pressure, increasing risk levels of hs-CRP, were in addition to abdominal obesity, also associated with triglycerides. Less patients with abdominal obesity reached the treatment targets recommended by the Swedish National Board of Health and Welfare for glycemic control (HbA1c ≤ 52 mmol/mol) and systolic blood pressure (≤ 130 mmHg), and no patients with abdominal obesity reached all treatment targets for TC, LDL, and blood pressure [22].

Strengths of our study are first that the population of patients with T1D was well-defined, since persons with severe comorbidities and severe substance abuse were excluded. Second, hs-CRP levels above 10 mg/l were excluded, and the CRP values were divided into 3 groups with low-, moderate- or high-risk for future cardiovascular events, as have been recommended in previous research [19]. Also, we performed a response analysis and explored whether persons with and without hs-CRP measurements differed. The patients with hs-CRP measurements had lower prevalence of abdominal obesity, otherwise they did not differ for any variable included in this study. Third, we explored interactions between the included metabolic variables.

The main limitation of our study was the rather small number of obese persons, particularly when gender sub analyses were performed. There are several possible type 2 errors. The association between the use of lipid-lowering drugs and abdominal obesity did not reach significance. The prevalence of both lipid-lowering drugs and anti-hypertensive drugs in patients with cardiovascular complications was high, but the associations were not significant. Second, the number of hs-CRP values measurements was limited, as we decided not to include hs-CRP measurements stored for more than 1 year. Despite the limited number of hs-CRP measurements and the lower prevalence of obesity in persons with hs-CRP measurements, the moderate and high-risk-levels of hs-CRP were strongly associated with abdominal obesity and triglyceride levels.

We have previously shown an association between alexithymia and abdominal obesity in this sample of patients with T1D [25]. In this study, we demonstrated the impact of abdominal obesity in T1D by the associations with cardiovascular complications, marked impaired glycemic control, low-grade inflammation, systolic blood pressure and triglycerides, all risk factors for future cardiovascular complications [1, 8–12, 14, 18]. We found a link between impaired glycemic control and raised triglycerides, which is in accordance with findings in patients with T2D [9]. Women with T1D are at a higher risk for atherosclerosis and cardiovascular death than men [1, 2]. One explanatory factor might be the noticeably higher prevalence of abdominal obesity in the women compared to the men with T1D, demonstrated in this study and in previous research [5, 25]. The prevalence of general obesity was almost twice as high in the women with T1D compared to women in the general Swedish population [7]. The reasons for the excessive abdominal obesity prevalence in women with T1D were not explained by this study, and further research of this subject is suggested. Apart from abdominal obesity, the only positive association with women was higher HDL, which is not a risk factor for cardiovascular disease according to previous research [13].

Another gender difference noted was that the men had higher blood pressure than the women, which is in accordance with previous research [34]. The prevalence of high systolic blood pressure (> 130 mmHg) was significantly higher in patients using anti-hypertensive drugs than in non-users, and the treatment target for systolic blood pressure was not obtained for a large proportion of persons using anti-hypertensive drugs. Weight reduction might help to reduce systolic blood pressure [35]. There is evidence that low-density lipoprotein (LDL) is a causal agent in the atherothrombotic process [8]. The use of lipid-lowering drugs is associated with a lower risk for cardiovascular disease and death [14]. Patients in this study using lipid-lowering drugs were successful in reaching the treatment goals for LDL more often than non-users. Improved treatment with lipid-lowering drugs is therefore suggested for patients with T1D and high LDL, in addition to weight reduction.

Due to the described detrimental effects of obesity in T1D, it is necessary to try new ways to both prevent and treat obesity. Reports of beneficial effects on weight and HbA1c have been reported for sodium-glucose cotransporter (SGLT2) inhibitors and glucagon-like peptide-1 (GLP-1) analogues [36]. Structured nutrition therapy, including reduced energy intake, lower total carbohydrate intake, and carbohydrates with lower glycemic index, has been recommended in combination with aerobic and resistance exercises [37]. As alexithymia was associated with abdominal obesity [25], psychoeducation aiming at increased emotional awareness could also be tried [28].

## Conclusions

Significant associations between abdominal obesity and both cardiovascular disease and cardiovascular risk factors were found in 284 patients with T1D. Low-grade inflammation, increased systolic blood pressure, inadequate glycemic control, and increased triglycerides were linked with abdominal obesity. The obesity prevalence was particularly high in women. Action against obesity is urgent to prevent cardiovascular complications in patients withT1D.

## Abbreviations

AHD: Anti-hypertensive drugs
AOR: Adjusted odds ratio
BMI: Body mass index
CI: Confidence interval
COR: Crude odds ratio
CSII: Continuous subcutaneous insulin infusion
GLP-1: Glucagon-like peptide-1
HDL: High-density lipoprotein
LDL: Low-density lipoprotein
LLD: Lipid-lowering drugs
MDII: Multiple daily insulin injections
OAA: Oral antidiabetic agents
SGLT2: Sodium-glucose cotransporter-2
T1D: Type 1 diabetes mellitus
TC: Total cholesterol
WC: Waist circumference
